# Transcript isoforms and alternative splicing in polyploid *Brassica napus* under heat and cold stress

**DOI:** 10.1093/aob/mcaf220

**Published:** 2025-09-11

**Authors:** Ryan E Bailey, Keith L Adams

**Affiliations:** Department of Botany, University of British Columbia, 6270 University Blvd., Vancouver, BC V6T 0A2, Canada; Department of Botany, University of British Columbia, 6270 University Blvd., Vancouver, BC V6T 0A2, Canada

**Keywords:** Alternative splicing, *Brassica napus*, canola, duplicated genes, gene expression, polyploidy, temperature stress, transcript isoforms

## Abstract

**Background and Aims:**

Polyploidization events have occurred many times during the evolution of flowering plants and have played a major role in genome evolution. Genes duplicated by polyploidy, termed homeologues, can diverge in function or new functions can evolve. There has been considerable interest in characterizing the transcriptomes of polyploid plants. One aspect of gene expression is alternative splicing (AS) by which precursor mRNAs are differentially spliced to form multiple mature mRNA isoforms. The effects of abiotic stress conditions on transcript isoform diversity of homeologues in polyploids has received little attention. *Brassica napus*, an allopolyploid derived from *B. rapa* and *B. oleracea*, is a model to study genetic complexities in polyploids. *Brassica napus* contains A homeologues that are derived from *B. rapa*, and C homeologues that are derived from *B. oleracea*.

**Methods:**

We conducted global analyses of transcript isoforms in *B. napus* using single molecule long-read sequencing of plants subjected to heat and cold stress treatments.

**Key Results:**

Cold stress reduced the number of isoforms produced by a given gene, whereas heat stress increased the number of isoforms. There was also a heat-responsive increase in the number of AS events. Heat stress induced a higher number of transcripts predicted to be probable targets of nonsense-mediated decay. C homeologues were more likely to produce more isoforms relative to A homeologues. A large proportion of homeologous pairs display shifts in their relative isoform distributions across homeologues in response to stress.

**Conclusions:**

Overall, our analyses reveal opposing shifts in isoform composition in response to cold and heat stress, as well as skewed isoform distributions across subgenomes. These results indicate that heat and cold stress can have considerable effects on the isoform composition of homeologous transcripts, which may help polyploids respond to temperature stresses.

## INTRODUCTION

Polyploidy, or whole genome duplication (WGD), has played a major role throughout the evolution of plants and has long been considered a major driver of evolution across a broad range of plant lineages (reviewed in Renny-Byfield and Wendel, 2014; Alix *et al*., 2017). Polyploidization events have occurred many times during the evolution of flowering plants (angiosperms). Polyploidy occurred at the base of angiosperms and thus all angiosperms have experienced at least one round of polyploidy in their evolutionary history (Jiao *et al*., 2011). Many angiosperm lineages have experienced additional polyploidy events during their evolutionary history. For example, analysis of the complete genome sequence of *Arabidopsis thaliana* revealed two recent WGDs (denoted α and β) which occurred within the Brassicaceae lineage and a triplication event (denoted as γ) which is likely to be shared by all eudicots (Blanc *et al*., 2003; Jiao *et al*., 2012). Regarding the role polyploidy plays in the evolution of angiosperm lineages, it has been estimated that roughly 15 % of angiosperm speciation events can be linked to polyploidy (Wood *et al*., 2009). Additionally, polyploidization has contributed to the evolution of novel functions such as the evolution of floral structures and increased disease resistance. Polyploidy has been implicated in adaptation to various environmental stressors (reviewed in Van de Peer *et al*., 2021; Tossi *et al*., 2022).

Polyploidization leads to a sudden and drastic increase in genetic material and a doubling of the entire gene set. This sudden proliferation of genetic material can lead to a cascade of consequences for the organism, in both the short and long term. Newly formed polyploids, termed neopolyploids, may undergo substantial genome reorganization, exchanges between genomes, gene loss and significant alterations in gene expression (reviewed in Adams and Wendel, 2005; Renny-Byfield and Wendel, 2014). Polyploidy results in a set of duplicated genes which are each contained within the respective parental genome of the polyploid, referred to as subgenomes. In allopolyploids, duplicate gene pairs are referred to as homeologous pairs, or homeologues. These duplicate pairs can undergo a multitude of fates, with some pairs being retained for millions of years (reviewed in Adams and Wendel, 2005). Several evolutionary fates for these duplicated genes exist, including (1) pseudogenization, in which one copy loses function or expression; (2) retention, in which both copies retain the original function or expression pattern; (3) neofunctionalization, in which one copy gains a new function or expression pattern; and (4) subfunctionalization, in which the original function of the expression pattern is subdivided between the duplicates.

Allopolyploids often display distinct patterns of gene regulation, gene expression, epigenetic differences and preferential gene loss across the subgenomes (reviewed in Adams, 2007; Madlung and Wendel, 2013; Nieto Feliner *et al.*, 2020). Homeologue expression biases are of particular importance in the study of polyploid evolution, as some plant species have demonstrated widespread transcriptional differences between homeologous gene pairs (e.g. Flagel *et al*., 2008; Buggs *et al*., 2011; Wang *et al*., 2016*b*; Khan *et al*., 2022). One important aspect of genomic bias across subgenomes is dominance at the level of gene expression, in which genes from one subgenome show consistently higher levels of expression relative to the other subgenome; this phenomenon is referred to as subgenome dominance (Yoo *et al*., 2013; Edger *et al*., 2017; Bird *et al*., 2020). Patterns of homeologous expression have been shown to be affected by abiotic stress conditions. In a study of hexaploid bread wheat, Liu *et al*. (2018) found differential expression of many homeologues in response to drought, heat, and drought plus heat stresses. Analysis of homeologous gene expression in *Brassica napus* revealed differential expression of homeologues and a consistent A_T_ subgenome bias in response to heat, cold and drought stresses (Lee and Adams, 2020).

Alternative splicing (AS) is a fundamental aspect of transcriptional regulation by which precursor messenger RNAs (pre-mRNAs) from multiexon genes are spliced to form mature messenger RNAs (mRNAs) which produce multiple mRNA isoforms. AS is thought to be a major contributor to the transcriptomic and proteomic complexity observed across a wide variety of organisms (Nilsen and Graveley, 2010). In plants, transcriptome-wide analysis has revealed that AS occurs extensively and it is estimated that over 60 % of intron-containing genes undergo AS (Reddy *et al*., 2013). AS events are categorized into five distinct types: (1) intron retention (IR) in which an intron is not spliced out of the transcript; (2) exon skipping (ES) in which a given exon is skipped and not retained in the transcript; (iii) alternative donor (ALTD) in which an alternative 5′ splice site is used; (4) alternative acceptor (ALTA) in which an alternative 3′ splice site is used; and (5) alternative position (ALTP) in which both alternative 5′ and 3′ spice junctions are used. Analysis of AS in several plant species has revealed that IR is the most predominant type of event with ES being the least common (reviewed in Reddy *et al*., 2013). A mechanism by which AS exerts transcriptional regulation in plants is nonsense-mediated decay (NMD), which can modify the relative expression dosage of a target gene if a given isoform undergoes an AS event that introduces a premature termination codon (Lewis *et al*., 2003). Research in *A. thaliana* has revealed that NMD plays a role in regulating expression levels of various genes including important regulatory genes involved in plant development (Kalyna *et al*., 2012).

In the context of polyploids, AS patterns can be divergent across subgenomes. This was first shown in a study of 74 homeologous gene pairs in *B. napus* (Zhou *et al*., 2011). More recent RNA-sequencing (RNA-seq) transcriptome studies of AS in homeologous genes in *B. napus*, wheat, cotton and synthetic rice polyploids have shown considerable subgenome AS divergence between homeologues (Liu *et al*., 2018; Wang *et al*., 2018; Lee and Adams, 2020; Yao *et al*., 2020; Yu *et al*., 2020; Gao *et al*., 2021). AS of homeologous genes has been examined in response to abiotic stress treatments. A study of hexaploid wheat found that homeologous genes exhibited differential AS responses under drought and/or heat stress, with homeologues belonging to the B subgenome exhibiting higher AS activity relative to the A and D subgenomes (Liu *et al*., 2018). In *B. napus*, Lee and Adams (2020) revealed extensive homeologue AS divergence and a C_T_-subgenome bias in AS under heat, cold and drought stress treatments.

Most studies of alternative splicing in polyploids have utilized short read sequencing (100–150 bp, typically with Illumina). However, that approach makes isoform reconstruction and thus isoform identification quite difficult and error-prone (Hardwick *et al*., 2019; Wang *et al*., 2019). In particular, AS events can introduce considerable ambiguities during this process as it can generate both divergent and/or partially redundant isoforms at a given gene locus (Steijger *et al*., 2013). This makes accurate detection of splicing isoforms difficult when using a short-read sequencing approach due to the inability of software packages to resolve these ambiguities in order to determine the actual combinations of splice-site usage. Long-read sequencing, however, removes these ambiguities by capturing entire transcripts in a single read, thus allowing researchers to obtain splicing isoforms directly without assembly (Sharon *et al*., 2013). This has been demonstrated in a study of maize in which researchers found that short-read assemblers such as Cufflinks and Trinity were only able to reconstruct small percentages (22 % and 8 %, respectively) of the isoforms that were discovered using PacBio long-read sequencing (Wang *et al*., 2016*a*, *b*).

The direct detection of isoforms using long-read sequencing is referred to as isoform sequencing (Iso-Seq). Iso-Seq has now enabled researchers to more accurately assess the role that AS plays in transcriptional regulation by offering a clear view of how individual AS events manifest as distinct isoforms. Iso-Seq has been used in several recent analyses to assess the role that AS plays in isoform diversity. In the first such study in polyploid plants, Wang *et al*. (2018) studied allopolyploid cotton (*Gossypium barbadense*) and discovered that over 50 % of homeologous gene pairs produce divergent isoforms in each subgenome, furthering our understanding and providing insights into the complexity of AS in polyploid species (Wang *et al*., 2018). In *B. napus*, Yao *et al*. (2020) used PacBio RSII to examine the complexity of isoforms, Rousseau-Gueutin *et al*. (2020) used Nanopore to identify AS events in the Darmor-bzh reference genome, and Li *et al.* (2022) used Nanopore with both natural and resynthesized *B. napus* to examine transcript isoforms. The application of Iso-Seq to uncover the complexities of AS in response to abiotic stress has not been extensively examined. In diploid *Populus trichocarpa*, Iso-Seq analysis revealed that abiotic stress can induce broad changes in isoform profiles including the regulation of certain IR events in a stress-specific manner (Filichkin *et al*., 2015). However, how isoform composition changes in polyploid plants in response to stress is unexplored.

*Brassica napus* (Brassicaceae) (AACC; oilseed rape) is a recently formed allopolyploid. The hybridization between its progenitors *B. oleracea* (CC: Mediterranean cabbage) and *B. rapa* (AA; Asian cabbage) occurred approximately 7500 years ago (Chalhoub *et al*., 2014). *Brassica napus* contains the A subgenome with A_T_ homeologues that are derived from *B. rapa*, and the C subgenome with C_T_ homeologues that are derived from *B. oleracea*. It represents a model to study aspects of AS and its effects on isoform diversity due to the availability of a high-quality reference genome and annotation, including the identification and annotation of homeologous gene pairs (Chalhoub *et al*., 2014).

There are numerous possible ways in which AS can exert changes in isoform diversity in polyploids in response to abiotic stress. First, the relative number of isoforms produced by a gene can vary in response to stress. Second, the relative number of isoforms that are produced by each member of a homeologous gene pair may be divergent in some pairs and the relative number of isoforms each homeologue contributes to the transcriptome may change in response to abiotic stress. Third, isoform structure(s) may diverge in response to stress or relative to the other duplicate gene in the homeologous pair. The goal of this study was to investigate the impact of AS on transcript isoform diversity in allopolyploid *B. napus* in response to heat and cold stresses, through the use of long-read sequencing technology. Specifically, we aimed to determine how the number and diversity of isoforms compares between the A and C subgenomes and how they respond to heat and cold stress treatments.

## MATERIALS AND METHODS

### Plant material and abiotic stress treatments

Plant growth and temperature stress treatments followed the protocol of Lee and Adams (2020). Seedlings of *B. napus* (cultivar ‘Sentry Summer Rape’; Rimmer *et al*., 1998) were grown in growth chambers with 16-h day/8-h night photoperiods at 22 °C, relative humidity of 50 % and a light intensity of 230–240 μmol m^−2^ s^−1^. After 4 weeks of growth, 15 healthy seedlings were selected for each condition (control, cold and heat). Seedlings in the cold treatment group were subjected to 4 °C for 24 h (Sangwan *et al*., 2001; Xiong *et al.*, 2013; Lee and Adams, 2020) and seedlings in the hot treatment were subjected to 35 °C for 24 h (Annamalai and Yanagihara, 1999; Young *et al*., 2004; Lee and Adams, 2020), and the control seedlings remained at 22 °C for 24 h. All other conditions were held constant. For each biological replicate the first true leaves were pooled from three individuals and three biological replicates were prepared for each condition. The selected leaflets were flash frozen in liquid nitrogen and stored at −80 °C until RNA extraction. Total RNA was extracted using the Ambion RNAaqueous kit and possible DNA contamination was removed using the Ambion Turbo DNA-free kit. The quantity and quality of the whole RNA were assessed using a spectrophotometer and through the inspection of the 23S and 16S rRNA bands using agarose gel electrophoresis. Preparation of cDNA with the SMARTer stranded RNA-seq kit according to the manufacturer’s instructions, and isoform sequencing with the Pacific Biosciences Sequel platform, were performed at Génome Québec (Montreal, Canada).

### Isoform structure analysis

Raw sequence data were processed into circular consensus sequences (CCSs) and full-length reads were detected and sorted using Pacific Biosciences’ IsoSeq3 pipeline (https://github.com/PacificBiosciences/IsoSeq). Full-length non-chimeric (FLNC) reads were then pooled from each of the biological replicates within each stress condition. FLNC reads were mapped to the *B. napus* reference genome using minimap2 with the parameters -ax splice -t 30 -uf -secondary = no -C5 (Chalhoub *et al*., 2014; Li, 2018). Identical mapped isoforms were then collapsed to obtain a final set of unique full-length isoforms using the python script collapse_isoforms_by_sam.py from the Cupcake-ToFU (Transcripts Isoforms: Full-length and Unassembled) python library (Tseng, 2019). Supplementary Data Figure S1 shows processing of raw isoform sequence data and alignment. Read counts for each stage are reported in Table S1.

Filtering of the dataset was completed to remove genes which had isoform counts that lie considerably outside of the known distribution of isoforms per gene based on the *B. napus* genome annotation (Chalhoub *et al*., 2014) and thus probably represent outliers. Outliers were identified using a *Z*-score approach in which *Z*-scores were calculated for each gene, and genes which reported a *Z*-score >3, representing data points which are 3s.d. above the mean, were removed from the dataset.

Structural characterization, splice junction analysis, coding potential and NMD prediction of full-length unique isoforms was performed using sqanti_qc.py from the SQANTI2 (Structural and Quality Annotation of Novel Transcript Isoforms) python library (Tardaguila *et al*., 2018). Downstream analysis, statistical analysis and graphical representation of the resultant output were performed in R.

#### Isoform characterization

Using the non-redundant set of isoforms produced by the shared set of 31 953 genes from each condition, the resultant isoform models were compared to the *B. napus* annotation provided by Chalhoub *et al*. (2014). Isoform characterization was determined by comparing the structures of each isoform to the known transcript models contained within the annotation. Each isoform was categorized into one of five categories: (1) full-splice match (FSM) – isoform matches the reference annotation perfectly; (2) incomplete-splice match (ISM) – isoform matches consecutive, but not all, splice junctions; (3) novel in catalogue (NIC) – isoform uses a novel combination of previously annotated splice junctions; (4) novel not in catalogue (NNC) – isoform uses at least one novel splice junction; and (5) genic genomic – isoform overlaps with introns and exons of another gene.

### Identification of stress responsive changes in isoform production

Log_2_ ratios were calculated from isoform counts per gene in the abiotic stress condition relative to isoform counts per gene in the normal condition. Each gene was categorized into one of three discrete categories: (i) decrease, log_2_ ratio ≤ −1; (ii) equal, log_2_ ratio between −1 and 1; and (iii) increase, log_2_ ratio ≥ 1. Functional annotation of genes within each category was completed as described in the section below.

### Alternative splicing detection

AS event detection was performed using the generateEvents command from the SUPPA2 pipeline using the parameters -i $INPUT_GTF -o $LIBRARY -f ioe -e SE SS MX RI FL (Alamancos *et al*., 2015). Events were generated using the GTF files generated with sqanti_qc.py. Downstream analysis and statistical tests of the resultant output were completed in R.

### Homeologue identification and comparison

Homeologous pairs were identified and sorted from the resultant SQANTI2 and SUPPA2 output for downstream analysis using the *B. napus* homeologue list provided by Chalhoub *et al.* (2014). Sorting and data manipulation were performed in R. Isoform diversity across a given homeologous pair was calculated following the method developed by Wang *et al*. (2018):


$$\mathrm{Lo}g_{2}\left(\frac{\mathrm{Number}\;\mathrm{of}\;\mathrm{isoforms}\;\mathrm{producted}\;\mathrm{by}\;\mathrm{the}\;A_{T}\;\mathrm{hom}\;\mathrm{eologous}\;\mathrm{gene}}{\mathrm{Number}\;\mathrm{of}\;\mathrm{isoforms}\;\mathrm{producted}\;\mathrm{by}\;\mathrm{the}\;C_{T}\;\mathrm{hom}\;\mathrm{eologous}\;\mathrm{gene}}\right)$$

The three categories were as follows: (i) C_T_ > A_T_, log_2_ ratio ≤ −1; (ii) A_T_ = C_T_, log_2_ ratio between −1 and 1; and (iii)) A_T_ > C_T_, log_2_ ratio ≥ 1. Downstream analysis and statistical tests of the resultant output were completed in R.

### Functional annotation

Gene ontology (GO) enrichment analysis was performed using the R package topGO (Alexa and Rahnenfuhrer, 2020). Genes which showed significant changes in isoform diversity in response to stress, homeologous pairs in each of the three groups listed above and homeologous pairs which changed categorization in response to stress were functionally categorized by their GO IDs using the reference genome and their *A. thaliana* orthologues. These orthologues were previously annotated by Chalhoub *et al.* (2014). Statistical tests of enrichment were done using Fisher’s exact test in R.

#### Reverse transcription-PCR verification of isoforms

Total RNA from the same samples was prepared, and reverse transcription (RT)-PCR was performed. A selection of genes across the control, hot and cold treatments were randomly selected and primers were designed to span alternative splicing events in order to generate distinct amplicons for each isoform detected in the sequence data. Reverse transcription was completed using Invitrogen Superscript III Reverse Transcription and subsequent PCR was completed on the resultant cDNA with the following conditions: initial denaturation (95 °C for 2 min), denaturation (95 °C for 20 s), annealing (55–57 °C depending on the primer set for 20 s) and extension (72 °C for 20 s), followed by a final extension (72 °C for 5 min); 35 cycles of the denaturation, annealing and extension were completed. RT-PCR products were visualized by gel electrophoresis (Supplementary Data Fig. S6).

## RESULTS

### *Brassica napus* isoform sequencing and read alignment

Iso-Seq was done with a total of nine samples: three biological replicates for each of the three conditions. This resulted in a total in a total of 3 015 152 FLNC reads following processing of the subreads and subsequent CCSs. Mapping accuracies of the uniquely mapped reads were 99.92, 99.94 and 99.91 % for normal, cold and hot, respectively (Supplementary Data Table S1).

### Isoform length and count distributions across conditions and subgenomes

**Fig. 1.**
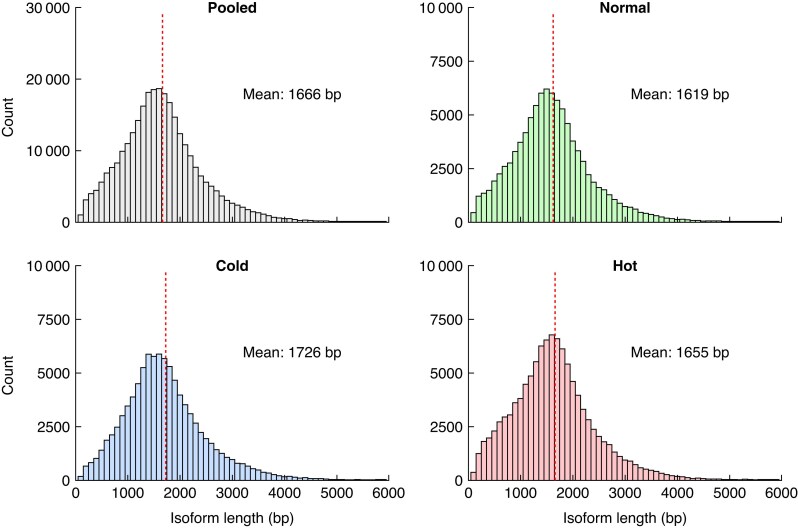
Distribution of isoform read lengths, pooled and separated by condition. Vertical dotted red line indicates the mean isoform length for each group.

A total of 32 449 genes were captured across all three conditions with a set of 31 953 genes shared by all three conditions (Supplementary Data Fig. S2) representing 98.5 % of the total genes captured. Only a small number of captured genes were unique to each condition (Fig. S2). To analysde differences across the A_T_ and C_T_ subgenomes (derived from the *B. rapa* and *B. oleracea* progenitors), we used a set of 8744 homeologous pairs for which both the A_T_ and C_T_ subgenomes were represented across all three conditions. Mean isoform lengths were 1619, 1726 and 1655 bp, for the normal, cold and hot conditions, respectively (Fig. 1). The interquartile ranges, representing isoforms for the central 50 % of the distribution, were tightly conserved across all three conditions with 1116–2003 bp for normal, 1206–2120 bp for cold and 1124–2057 bp for hot.

A unique set of non-redundant isoforms for each condition was identified. A total 98 861 isoforms were detected from 32 268 genes in the normal condition, resulting in an average of 3.06 isoforms per gene. For the cold condition, a total of 96 965 isoforms were detected from 32 212 genes, resulting in an average of 3.02 isoforms per gene. For the hot condition, 111 976 isoforms were detected from 32 233 genes, resulting in an average of 3.47 isoforms per gene, the highest of all three conditions (Supplementary Data Table S2).

**Fig. 2.**
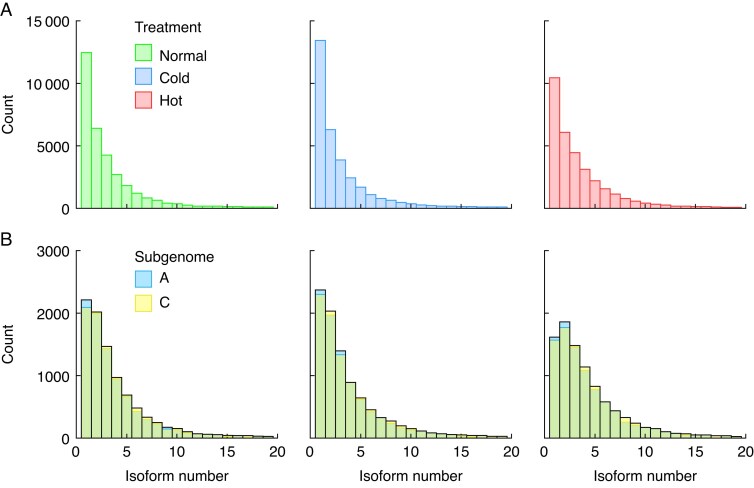
Distributions of isoforms per gene count. (A) Across abiotic conditions (*n* = 31 953), and (B) across subgenomes. Blue represents the A_T_ subgenome, yellow represents the C_T_ subgenome and green represents where the distributions overlap.

To detect differences across abiotic stress conditions relative to the control (normal) condition, the distribution of isoform counts per gene across the shared set of 31 953 genes was examined. Globally across the shared set of 31 953 genes, each condition showed a similar distribution, with genes that produce a single isoform at the highest frequency. As the number of isoforms per gene increases, the frequencies drop steadily (Fig. 2A). Similar trends are also seen in the distributions across subgenomes, using the shared set of 8744 homeologous genes (Fig. 2B). Subgenomic distributions are tightly conserved across all three conditions. Genes that produce two isoforms show the highest frequency in the hot condition subgenomic distribution (Fig. 2B).

Supplementary Data Table S3 summarizes the number of isoforms produced from the shared set of 8744 homeologous genes across the subgenomes in all three conditions as well as the isoform per gene calculations. In each condition there is a slight and consistent increase towards the C_T_ subgenome in the number of isoforms produced by each gene.

### Isoform characterization reveals previously unidentified splice junctions

Isoforms were categorized into five categories (see Methods) and the distributions of these categories are visualized in Supplementary Data Fig. S3. Total counts for all categories are reported in Table S4. The distributions of the categorizations across all three conditions remain conserved. The categories which represent isoforms containing previously annotated splice junctions and thus match the reference closely (FSM, ISM and NIC) represent roughly 51–52 % of the isoforms detected across all three conditions, whereas isoforms containing at least one novel junction (NNC) represent 37–39 % of the isoforms detected across all three conditions (Table S4, Fig. S3). The NNC category represents the largest percentage of isoforms across any individual category for each condition. Thus, our Iso-Seq approach has facilitated the discovery of a large number of previously unknown isoforms.

### Stress responsive changes in isoform production result in skewed distributions in response to abiotic stress

**Fig. 3.**
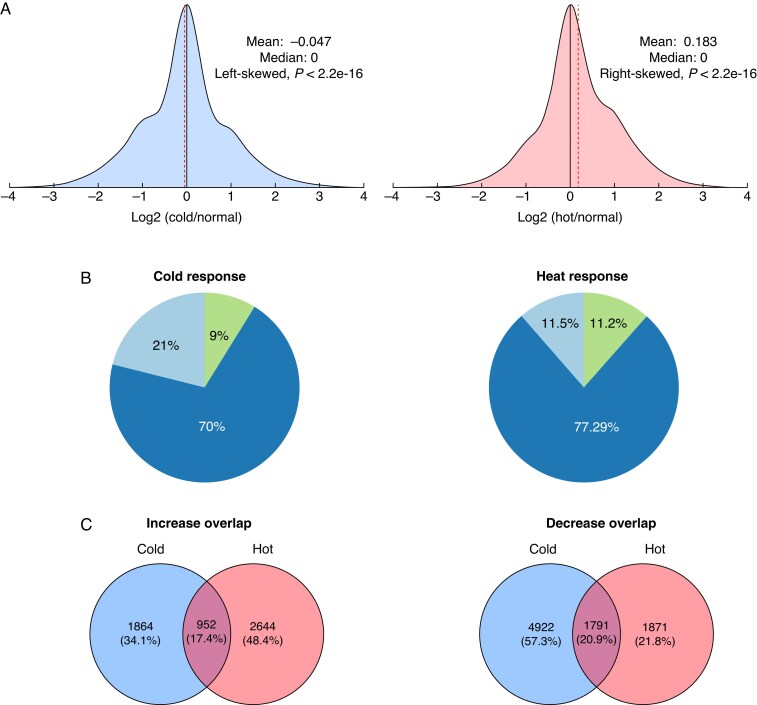
(A) Density plot of the log_2_ ratios of isoforms produced in the abiotic stress condition relative the normal condition. The cold response distribution is left-skewed (test of symmetry, left-skewness, *P* < 2.2 × 10^–16^). The heat response distribution is right-skewed (test of symmetry, right-skewness, *P* < 2.2 × 10^–16^). Vertical dotted red line represents the mean. (B) Distributions of isoforms across three categories (decrease: log_2_ ratio < −1, equal: log_2_ ratio between −1 and 1, increase: log_2_ ratio >1). (C) Overlap of the genes which show both an increase and a decrease in isoforms produced in response to each abiotic condition.

To examine whether there was a stress responsive shift in isoform profiles in our abiotic stress conditions, we examined the log_2_ ratios of isoforms produced in the abiotic stress conditions relative to the normal condition across the shared set of 31 953 genes. We found that in response to both cold and heat stress, the distributions of the log_2_ ratios were asymmetric (Fig. 3A). The distribution of the cold-responsive log_2_ ratios showed a negatively skewed, or left-skewed, distribution (test of symmetry from Miao *et al*., 2006; *P* < 2.2 × 10^−16^). For the heat-responsive distribution, the opposite was observed in which log_2_ ratios were significantly skewed to the right, or positively skewed (test of symmetry, *P* < 2.2 × 10^−16^) (Fig. 3A). These results indicate fewer isoforms are produced per gene in response to the cold treatment, thus shifting the log_2_ ratio distribution towards the negative direction. In contrast, there is an overall increase in the number of isoforms produced per gene in response to heat, as shown by the shift in the positive direction.

To further examine these distributions, genes were sorted into three discrete categories based upon their log_2_ ratio (Fig. 3B; Table 1). Genes in the ‘decrease’ category correspond to genes which produce a log_2_ ratio ≤ −1, genes in the ‘equal’ category represent genes which produce a log_2_ ratio between −1 and 1, and genes in the ‘increase’ category correspond to genes producing log_2_ ratios ≥ 1. Generally, the vast majority of genes in either stress response were placed in the ‘equal’ category representing 70 and 77 % of all genes in the cold response and heat response, respectively, suggesting that the majority of genes experience a relatively equally distributed isoform distribution in response to stress (Fig. 3B). However, the negatively skewed distribution in the cold response is also evident in the proportions of genes in each of the other categories: 21 % in the ‘decrease’ category and 9 % in the ‘increase’ category (Fig. 3B). Comparisons of these categories in the heat response showed 11.46 % in the decrease and 11.25 % in the increase category. This is probably due to the fact that the deviations in the log_2_ ratios responsible for the positively shifted log_2_ distribution occur within the bounds of −1 and 1; in other words, the shift occurs more centrally in the distribution rather than at the tails.

**Table 1.** Counts of genes which undergo large shifts in the number of isoforms produced in response to cold and heat treatments.

	Cold	Hot
Decrease	6713	3662
Equal	22 424	24 695
Increase	2816	3596

Next, we examined the degree of overlap among genes in the ‘decrease’ or ‘increase’ categories across both stress responses (Fig. 3C). A relatively small proportion of the genes were given the same categorization (increase or decrease) in both stress responses (17.4 % for increase, 20.9 % for decrease), thus indicating that the genes which undergo isoform profile shifts are largely unique to each stress response.

GO analysis was performed on the set of genes in each category across each stress type to predict possible enriched functions. The most significantly enriched function in the cold response: increase category was ‘response to abscisic acid’ (GO:0009737), a known regulator of the abiotic stress response in plants (Supplementary Data Table S5a). This suggests that genes involved in the abscisic acid (ABA) pathway undergo an increase in the number of isoforms produced by each gene in response to cold. In the cold response: decrease category, several of the enriched terms pertain to chloroplast localization, including ‘chloroplast stroma’ (GO:0009570), ‘chloroplast envelope’ (GO:0009941) and ‘chloroplast thylakoid membrane’ (GO:0009535) (Table S5c). This suggests that genes whose gene products are localized to the chloroplast experience an overall decrease in their isoform repertoires when exposed to cold stress. In the cold response: equal category, the most enriched term was ‘cytosol’ (GO:0005829) (Table S5c). In the heat response increase: category, ‘unfolded protein binding’ (GO:0051082) was among the most enriched terms, suggesting that genes which undergo an increase in the number of isoforms produced in response to heat stress are enriched for functions which may respond to proteins that have been denatured by the heat stress (Table S5b). In the heat response: decrease category, ‘DNA-binding transcription factor activity’ (GO:0003700) was shown to be among the most enriched terms, but the significance level was not comparatively high relative to the scores obtained in the cold response sets (Table S5b). As with the cold response: equal category, ‘cytosol’ (GO:0005829) was the most enriched term in the hot response: equal category, thus indicating that genes which maintain equally distributed isoforms in response to both abiotic stresses are commonly localized to the cytosol (Table S5c).

### Persistent increase in AS in response to heat and slight C_T_ increase across all three conditions

**Fig. 4.**
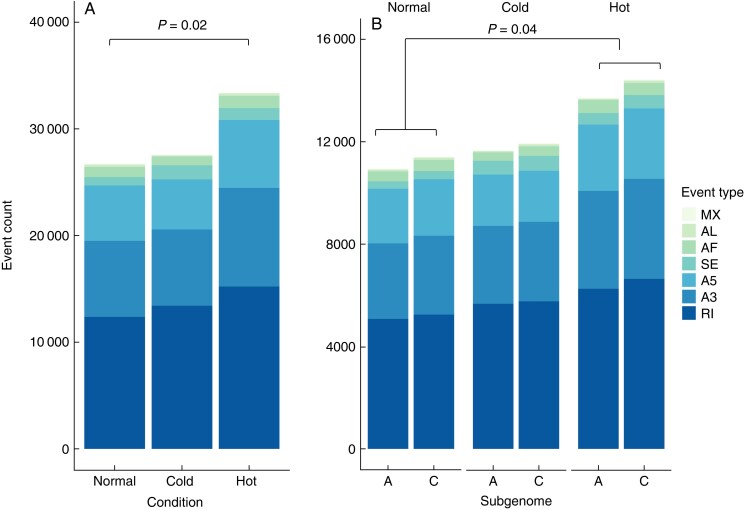
(A) AS events detected across 31 953 genes shared by each condition. A significant difference between the number of alternative splicing events in the normal condition and the hot condition was detected (Wilcoxon signed rank test, *n* = 7 categories of alternative splicing events, *P* = 0.02). (B) AS events detected in the 8744 homeologous pairs, separated by subgenome. There was a significant difference in cumulative AS events across both subgenomes in the response to heat (Wilcoxon signed rank test, *n* = 7 categories of alternative splicing events; *P* > 0.05). No significant differences were detected across subgenomes. See Table 2 for names of event types.

To further examine the dynamics resulting in the skewed isoform distributions, we detected and classified AS events across each condition and between subgenomes. We detected a significant increase in AS events in response to heat stress (Fig. 4A) (Wilcoxon signed rank test, *P* = 0.02), but not for cold stress (Fig. 4A) (Wilcoxon signed rank test, *P* = 0.94). Across each of the conditions, distributions of AS events remained relatively conserved (Table 2), with IR being the most abundant category at 46.2, 45.7 and 48.6 % for normal, cold and hot, respectively (Table 2). The second most abundant category was alternative 3′ splice site, representing 26.9, 27.6 and 26.0 % for normal, cold and hot, respectively (Table 2). The distributions of AS event types remain conserved, suggesting that although abiotic stress may result in a stress-responsive shift in the total number of events, the distribution of total events across each of the AS categories remains largely unaffected. The significant increase in total events in response to heat stress aligns with our previous observation that the log_2_ ratio of isoforms produced in the hot condition relative to the normal condition distribution is positively skewed. This skew is probably due to the increased AS activity generating more isoforms per gene following exposure to heat.

**Table 2.** AS event counts and percentages across abiotic conditions per subgenome (*n* = 8744).

	Normal	Cold	Hot
Exon skipping (SE)	781 (2.9 %)	1341 (4.9 %)	1153 (3.5 %)
Mutually exclusive exon (MX)	35 (0.1 %)	48 (0.2 %)	52 (0.16 %)
Alternative 5′ splice site (A5)	5191 (19.5 %)	4668 (16.9 %)	6359 (19.0 %)
Alternative 3′ splice site (A3)	7170 (26.9 %)	7169 (26.0 %)	9200 (27.6 %)
Intron retention (RI)	12 332 (46.2 %)	13 407 (48.6 %)	15 251 (45.7 %)
Alternative first exon (AF)	972 (3.6 %)	813 (3.0 %)	1139 (3.4 %)
Alternative last exon (AL)	202 (0.8 %)	113 (0.4 %)	227 (0.7 %)
Total	26 683	27 579	33 381

We conducted the same analysis using the set of 8744 homeologous pairs shared across all three conditions to examine the dynamics across the subgenomes. In each condition, there was no significant difference in numbers of AS events between the C_T_ subgenome relative to the A_T_ subgenome [Wilcoxon rank sum test, *P* = 0.71 (normal), *P* = 0.80 (cold), *P* = 0.71(hot)] (Fig. 4B; Table 3]. Comparing the cumulative AS event counts for both the A_T_ and C_T_ subgenomes, there is a significant increase in response to heat (Wilcoxon signed rank test, *P* = 0.04), but not for cold (Wilcoxon signed rank test, *P* = 0.57). The persistent significant signal for increased AS in response to heat across both sets of genes further supports the idea that AS is probably heat-responsive. AS event profiles of the various event types again show a high degree of conservation across stress types and subgenomes. The distributions also closely reflect the AS profiles reported in the global analysis above (Table 3), with IR being the most abundant category across conditions and between subgenomes (Table 3). Summaries of the counts of alternatively spliced genes and the average AS event counts per gene are reported in Supplementary Data Tables S6 and S7.

**Table 3.** AS event counts and percentages across abiotic conditions (*n* = 31 953 genes).

	Normal		Cold		Hot	
	A_T_	C_T_	A_T_	C_T_	A_T_	C_T_
Exon skipping (SE)	297 (2.7 %)	327 (2.9 %)	552 (4.73 %)	585 (4.9 %)	444 (3.2 %)	516 (3.6 %)
Mutually exclusive exon (MX)	12 (0.10 %)	17 (0.1 %)	15 (0.1 %)	23 (0.2 %)	21 (0.2 %)	22 (0.2 %)
Alternative 5′ exon (A5)	2216 (19.5 %)	2209 (19.3 %)	1983 (17.0 %)	1982 (16.6 %)	2589 (18.9 %)	2748 (19.1 %)
Alternative 3′ exon (A3)	2934 (26.9 %)	3068 (26.9 %)	3032 (26.0 %)	3097 (26.0 %)	3816 (27.8 %)	3909 (27.1 %)
Intron retention (RI)	5099 (46.7 %)	5264 (46.2 %)	5703 (49.0 %)	5783 (48.5 %)	6272 (45.8 %)	6646 (46.12 %)
Alternative first exon (AF)	375 (3.4 %)	427 (3.7 %)	333 (2.9 %)	385 (3.2 %)	510 (3.7 %)	482 (3.3 %)
Alternative last exon (AL)	73 (0.7 %)	90 (0.8 %)	30 (0.3 %)	70 (0.6 %)	52 (0.8 %)	85 (0.6 %)
Total	10 925	11 402	11 648	11 925	13 704	14 408

#### Homeologous pairs exhibit transcriptome-wide increases in C_T_ isoforms

**Fig. 5.**
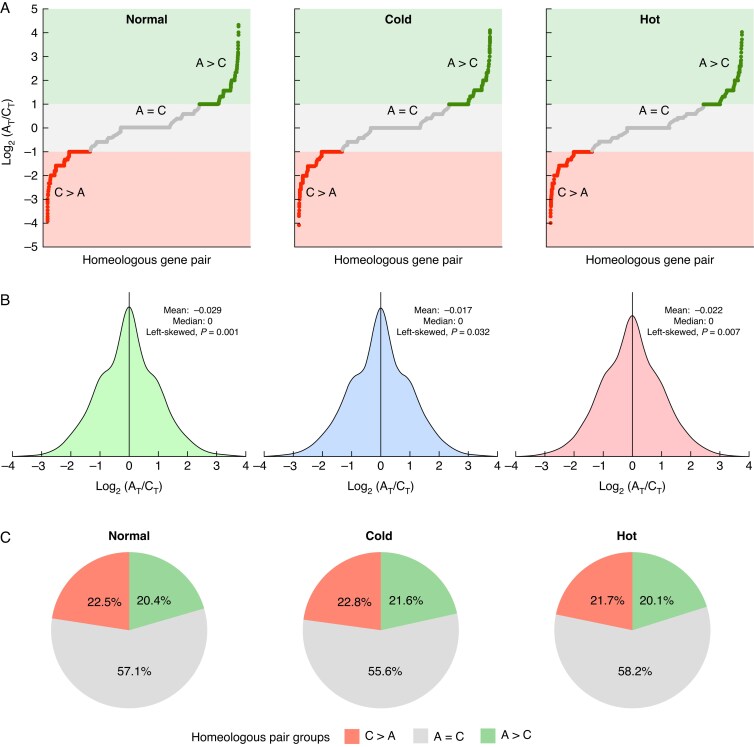
(A) Log_2_ ratio of isoform numbers of homeologous genes in A_T_ and C_T_ subgenomes, separated by condition. All gene pairs are categorized into three difference groups: C > A (log_2_ ratio ≤ −1), A = C (log_2_ ratio > −1, and log_2_ ratio <1), and A > C (log_2_ ratio ≥1). (B) Density plots of the log_2_ ratios for each condition (green = normal, blue = cold, red = hot); mean, medians and the results of the symmetry analysis are listed on the graphs. (C) Percentages of homeologues belonging to each of the homeologous pair groups, separated by condition.

To further explore post-transcriptional dynamics across the A_T_ and C_T_ subgenomes, we categorized each of the 8744 homeologous gene pairs based upon their isoform distribution across both subgenomes (see Methods for categories). The distributions and density plots are shown in Fig. 5A and B. Across the 8744 homeologous pairs, a range of log_2_ values are observed for each condition, indicating both isoform divergence and isoform conservation, in terms of isoform counts, depending on the particular homeologous pair. Next, we examined if any of the distributions, across conditions, exhibit skewness. The presence of skewness in the distributions will indicate whether homeologues from a certain subgenome consistently produce more isoforms relative to the homeologue from the opposite subgenome. In each of the distributions the mean is observed to be less than the median [median = 0 for all conditions, mean = −0.029 (normal), −0.017 (cold), −0.022 (hot)] (Fig. 5B). We find that, across all three conditions, the distributions show left-skewness, meaning the C_T_ homeologues are more likely to produce a greater number of isoforms relative to their A_T_ homeologues and this pattern remains conserved in response to both stresses [test of symmetry, left-skewness, *P* = 0.001 (normal), *P* = 0.032 (cold), *P* = 0.007 (hot)] (Fig. 5B). These skewed distributions are also evident when we compared the distributions of counts associated with each of the three categories across stress types (Fig. 5C). There is a consistent, yet slight, enrichment for homeologous pairs categorized as C_T_ > A_T_ relative to those categorized as A_T_ > C_T_, reflecting the left-skewed distributions identified above. C_T_ > A_T_ homeologous pairs represent 21.7–22.7 % of the homeologous pairs, and 20.1–21.6 % were categorized as A_T_ > C_T_ homeologues (Fig. 5C). In each condition the majority of pairs (55.6–58.2 %) were categorized as A_T_ = C_T_ (Fig. 5C).

Next, we determined whether the log_2_ distributions from either abiotic stress condition were independent from the distribution observed in the normal condition. We uncovered a significant difference in the heat stress distribution at a significance threshold of *P* < 0.05 (two-sample Kolmogorov–Smirnov test, *P* = 0.013). However, we did not detect a significant deviation from the normal distribution in response to cold stress (two-sample Kolmogorov–Smirnov test, *P* = 0.227). These results indicate that heat stress significantly perturbs the distribution of isoforms generated across homeologous pairs, but cold stress does not. In addition, the perturbation observed in the heat response, and lack thereof in the cold response, does not affect the consistent C_T_ > A_T_ trend of the distributions in each condition.

Finally, we conducted a GO enrichment analysis. Across both the C_T_ > A_T_ and A_T_ > C_T_ categories, few functional categories were consistently enriched across all three conditions (Supplementary Data Table S8). However, in the A_T_ = C_T_ category, ‘mRNA binding’ (GO:0003729) was among the most highly enriched functions for the normal, cold and hot conditions (Table S8b). Consistent enrichment in the A_T_ = C_T_ category suggests that homeologous pairs involved in mRNA binding are more likely to show a conserved repertoire of isoforms across a homeologous pair rather than a divergent repertoire.

### Homeologous isoform repertoire shifts in response to cold and heat stress

**Fig. 6.**
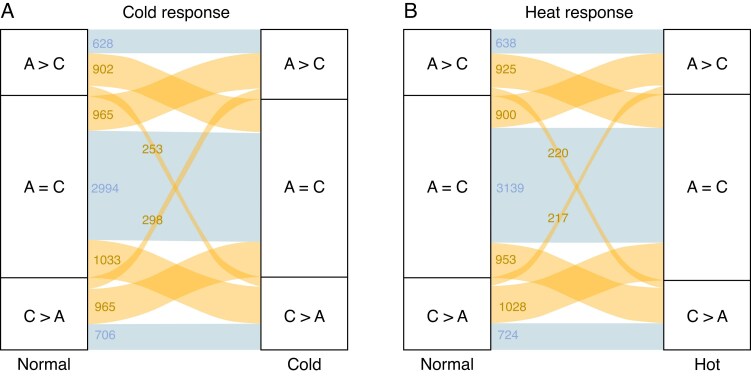
Changes in A_T_ and C_T_ isoform distribution categories in response to stress. Yellow alluvial flow represents homeologues that change categorization in response to stress, and blue alluvial flow represents homeologues that do not change categorization in response to stress across the 8744 homeologous pairs. (A) Cold response. (B) Heat response.

We detected stress-responsive changes in the categories for each of the 8744 homeologous pairs. We found that 50.5 and 48.5 % of homeologous pairs tested in the cold and heat response, respectively, exhibited a category change in response to abiotic stress, whereas 49.5 and 51.5 % of homeologous pairs tested in the cold and heat, respectively, did not (Fig. 6; Supplementary Data Table S9). Among homeologous pairs that exhibit a stress-responsive category shift, one of the most common shifts observed are pairs that move from the C_T_ > A_T_ category in the normal condition to the A_T_ = C_T_ category in the stress condition (11.6 % for cold and 11.0 % for heat stress) (Table S9). The least common category shifts were shifts between the C_T_ > A_T_ and A_T_ > C_T_ categories, and vice versa, as represented by the narrowest yellow alluvial flows in Fig. 6. The patterns observed in the frequencies associated with each of the category shifts in response to cold and heat stress are tightly conserved (Wilcoxon signed rank test, *P* > 0.99). The similarity in the alluvial flow patterning depicted in each panel of Fig. 6 demonstrates this. Interestingly, a seemingly concomitant ‘switching’ is observed between category shifts across both conditions. The proportions of homeologous genes which undergo a given switch (i.e. C_T_ > A_T_ → A_T_ = C_T_) are consistently similar to the opposite, or reversed, category shift (i.e. A_T_ = C_T_ → C_T_ > A_T_) (Table S9). This applies to shifts between the A_T_ = C_T_ category and the C_T_ > A_T_ or A_T_ > C_T_ categories, across both conditions (Fig. 6). Overall, these results indicate that a large proportion of homeologous pairs display shifts in their isoform distributions across homeologues, indicating the complex dynamics of the polyploid transcriptome.

We next determined if the genes identified in the category shifts were enriched for certain functions using GO analysis. We see similarly enriched functions across the various categories for both the cold and heat stresses (Supplementary Data Table S10). For the A_T_ = C_T_ → A_T_ = C_T_ category, which includes homeologous pairs which are seemingly resistant to stress-induced shifts in isoform repertoires, we again see an enrichment for ‘mRNA binding’ (GO:0003729) (Table 10b). Similar enrichment for terms pertaining to the chloroplast are observed in each stress type for this category as well, including ‘chloroplast thylakoid membrane’ (GO:0009535) and ‘chloroplast envelope’ (GO:0009941). Interestingly, ‘apoplast’ (GO:0048046) is also enriched across the cold and heat responses for the A_T_ = C_T_ → A_T_ = C_T_ shift (Table 10b). Despite the highly similar patterning of category shifts across both stress responses shown in Fig. 6, there was little conservation in the enriched functions for the homeologous pairs which move away from an equally distributed isoform distribution, in response to stress (Table S10). For example, the top enriched term for the A_T_ = C_T_ → C_T_ > A_T_ category shift in the cold response was ‘microtubule bundle formation’ (GO:0001578), but in the hot response it was ‘microtubule severing’ (GO:0051013), a seemingly opposite function (Table 10a).

### Analysis of known cold and heat responsive genes

To further examine the isoform repertoires in response to cold and heat treatments, we visualized the isoform profiles of homeologous pairs that are known cold and heat responsive genes. Two examples are shown in Supplementary Data Fig. S4. The homeologous pair BnaA03g38950 (A_T_ homeologue) and BnaC03g45990 (C_T_ homeologue), homologous to the cold response (COR) gene AT2G15970 in *A. thaliana*, show partially divergent isoform profiles in response to cold stress and across subgenomes (Fig. S4A). Interestingly, in the normal condition BnaA03g38950 lacks the first exon and part of the second exon, each of which are fully captured within the open reading frame (ORF) when exposed to cold stress. Additionally, these first two exons are intact in the majority of isoforms produced in the C_T_ homeologue (BnaC03g45990) with only one isoform (PB.4685.4) showing divergent intron/exon structures within the ORF. Notably, both the 5′ and 3′ untranslated regions (UTRs) vary across each homeologue and condition. The homeologous pair BnaA06g07260 (A_T_ homeologue) and BnCnng18070 (C_T_ homeologue), which is homologous to heat shock protein AT1G11660 in *A. thaliana*, is visualized in Fig. S4B. In the A_T_ homeologue, an overall increase in isoforms occurred in response to heat stress, with divergent structures produced by subtle changes in the 5′ splice site of the first exon, as well as a premature termination codon in isoform PB.10401.2 in the 7th exon. In the C_T_ homeologue, an opposite change is seen in response to heat in which fewer isoforms are produced (Fig. S4B).

### Incidence of predicted NMD increases in response to heat stress

To examine whether the AS patterns discussed in earlier sections might lead to NMD, we categorized isoforms based on whether a premature termination codon (PTC) was detected within its ORF. We found that the vast majority (82–84 %) of isoforms are unlikely to be targets of NMD (Supplementary Data Fig. S5A). However, slight differences in the relative frequencies of each NMD prediction are noted between the normal and heat conditions (Fig. S5A). The frequencies of isoforms in each of the prediction categories varies significantly in response to heat (Fisher’s exact test, *P* = 4.6 × 10^−7^), indicating a relationship between exposure to heat stress and an increase in the proportion of isoforms predicted to be likely targets of NMD. We did not detect a similar relationship in response to cold stress (Fisher’s exact test, *P* = 0.515). Looking at these patterns across the subgenomes (Fig. S5B), we can see that these frequencies are tightly conserved. Comparisons of the frequencies across the subgenomes in each condition reveal no significant differences; in other words, the subgenomic origin of a given isoform and its NMD prediction are independent [Fisher’s exact test, *P* = 0.754 (normal), *P* = 0.287 (cold), *P* = 0.218 (hot)] (Fig. S5B).

## DISCUSSION

### Abiotic stress effects on isoform distributions

Through our use of long-read sequencing we have generated a comprehensive isoform-level transcriptome of *B. napus* leaves from seedlings subjected to heat and cold stress treatments as well as untreated plants. This analysis facilitates our understanding of the complexities of this allopolyploid transcriptome, including how isoform distributions respond to temperature stress.

This study revealed that both cold and heat stress can shift isoform composition. When plants were exposed to cold stress, genes were more likely to produce fewer isoforms, as detected by a significant shift in the log_2_ ratio of isoforms produced in the cold condition relative to the normal (untreated) condition. Conversely, when exposed to heat stress, isoform composition shifted in the opposite direction, as genes were more likely to produce a greater number of isoforms when exposed to heat relative to the normal condition. The observed isoform composition shift detected with the heat stress mirrors what was detected in our AS event analysis. We found that exposure to heat significantly increases the number of AS events. Despite our detection of a shifted isoform distribution in response to cold stress, no significant differences were detected in the number of AS event counts in response to cold stress. Despite the observed heat-responsive increases in the number of AS events, the relative frequencies of AS event types are highly conserved; in other words, no particular event type seems to be enriched in response to heat stress.

Aspects of transcriptional regulation in *B. napus* have been shown to be heat stress-dependent (reviewed in Kourani *et al.*, 2022). In their global transcriptomic analysis, Lee and Adams (2020) demonstrated overall gene expression decreases across cold, heat and drought stresses, with cold stress having the greatest impact. Interestingly, whereas the cold stress showed overall gene expression decreases, AS increased in response to the cold stress, thus representing an inverse relationship between gene expression and AS (Lee and Adams, 2020). Evidence for such a relationship has also been obtained in polyploid wheat (Liu *et al*., 2018). Although we did not detect a similar cold-responsive increase in AS events in our long-read dataset, the observation of AS event counts being inversely correlated to gene expression raises the question of whether the characteristic stress-induced isoform composition shifts we observed in this study are paired with relative increases or decreases in overall expression of these isoforms. Other studies have made similar suggestions that AS responses to stress are independent relative to gene expression, showing that genes which are differentially spliced in response to stress are unlikely to be identified as differentially expressed genes (Ding *et al.*, 2014; Dong *et al*., 2018).

Across other species, AS has also been shown to change in a stress-dependent manner, foe example salt stress in *Arabidopsis* (Ding *et al*., 2014) and heat stress in grape (Jiang *et al*., 2017). However, the effects of abiotic stress on the actual isoform composition have remained understudied. In *Populus trichocarpa*, several abiotic stresses (heat, cold, drought and salinity) perturbed isoform profiles (Filichkin *et al*., 2018). These studies, in conjunction with our own, provide emerging evidence for the role that AS can play in modulating the transcriptome in a stress-specific manner and that stress-mediated AS regulation may be distinct from gene expression levels.

Regarding the molecular mechanism by which AS could modulate an abiotic stress response, and possibly contribute to the shifts described above, emerging evidence has pointed to the role that splicing factors may play in modulating the stress response through their targeting of components of the ABA pathway, a central component of the abiotic stress response (Laloum *et al*., 2018). Several splicing regulators have been reported to affect ABA sensitivity, providing further evidence that AS is an important mediator in the post-transcriptional regulation of plant stress responses (Papp *et al*., 2004; Lee *et al*., 2006; Kim *et al*., 2007; Zhang *et al*., 2011). Furthermore, studies have shown that plants treated with splicing inhibitors display responses that mimic stress signals in plants leading to the activation of ABA-inducible promoters and stomatal closure (AlShareef *et al*., 2017; Ling *et al*., 2017). Seemingly, ABA signalling, and thus the abiotic stress response, is fine-tuned through splicing activity among the genes involved within the ABA pathways. Within our dataset, genes which showed a large increase in the number isoforms in response to cold stress were significantly enriched for functions that relate to ABA signalling. Thus, genes involved in ABA signalling during the cold stress response undergo increased amounts of alternative splicing leading to a diversified set of isoforms in response to an abiotic stress, providing further evidence for the adaptive role AS may have in fine-tuning the ABA pathways in order to adapt to environmental stress.

### Subgenome isoform distributions change upon stress treatments

As part of our analysis of homeologous gene pairs, we determined whether the isoform count distributions across the pairs changed in a stress-responsive manner. We found that a large proportion of homeologous pairs do not retain their subgenome bias when subjected to either cold or heat stress. Interestingly, the subgenomic origin of the AS events responsible for a category shift are highly dynamic, and a given stress-responsive category shift could be the result of opposing AS events across both subgenomes. In other words, if a homeologous pair changes to having a larger set of C_T_ isoforms compared to A_T_ isoforms (i.e. A_T_ = C_T_ → C_T_ > A_T_) this could be a result of an increased number of AS events in the C_T_ subgenome and reduced number of AS events in the A_T_ subgenome. This underlines the highly complex nature of AS dynamics at work in the polyploid.

The detectable C_T_ subgenome skew uncovered in our analysis is somewhat analogous to the C_T_ subgenome bias in the extent of AS under three abiotic stress treatments detected by Lee and Adams (2020). While they were able to discover increased levels of AS in the C_T_ subgenome, we showed that a more numerous set of isoforms is generated from the C_T_ subgenome. Cases in which a certain subgenome exhibits more AS relative to another is not a phenomenon unique to *B. napus*. In their analysis of hexaploid wheat, Liu *et al*. (2018) found that the genes belonging to the B subgenome displayed higher counts of stress-induced AS events relative to the A and D subgenomes.

Although there are other aspects of transcriptional regulation not assessed in this study, our findings indicate that the C_T_ sub-transcriptome generated by the C_T_ homeologues contains more isoforms and thus may be more complex. Similar work in allopolyploid cotton has assessed the relative isoform distributions across subgenomes and has found that slightly more pairs produce more isoforms from the A_T_ homeologue than the D_T_ homeologue, relative to pairs showing the opposite distribution (Wang *et al*., 2018). However, they found that the majority of pairs tested did not show extreme differences in the number of isoforms generated by the two subgenomes, similar to our findings. Furthermore, our work adds to the emerging evidence that homeologous genes in allopolyploids show divergent counts of splicing isoforms. Divergent isoform counts across the subgenomes might lead to subfunctionalization or neofunctionalization, wherein new splice isoforms may provide the opportunity for new functions to evolve between the duplicates or possibly the original function of the homeologous pair to be subdivided between the two homeologues.

### Stress-responsive NMD

We uncovered a significant shift in the proportion of isoforms predicted to be likely targets of NMD in response to heat stress. Our detection predictions were based on whether the resultant isoform had a PTC within the ORF. If that criterion was met, the isoform was categorized as a likely target of NMD, and if not, it was designated as an unlikely target. This methodology does not include AS events in the 5′ and 3′ UTRs which sometimes result in transcript degradation by NMD (Drechsel *et al*., 2013), and thus it is not a perfect categorization of targets vs. unlikely targets of NMD. For heat stress, we are able to piece together our analysis of a stress-responsive increase in AS to a positively shifted isoform distribution, wherein plants under heat stress produced a higher number of isoforms per gene, to the heat-responsive increase in likely NMD targets. Despite this connection, we are unable to trace a similar line through the cold response, or across the subgenomes, where we can detect differences in isoform distributions across conditions or between subgenomes. Unlike the heat stress, our skewed stress-responsive and subgenomic isoform distributions do not manifest skewed, or shifted, proportions of likely or unlikely NMD targets. In other words, within our dataset, NMD does not act in an independent manner across subgenomes or in response to cold.

The detection of a stress-responsive signal in the increase of NMD candidates provides further evidence for the role that AS-mediated NMD (AS-NMD) may play in an adaptive stress response. Cases of stress-responsive NMD have been discovered in plants. For example, in their analysis of the NMD factor UPF3 in *Arabidopsis*, involved in the proper maintenance of NMD homeostasis, Vexler *et al*. (2016) revealed that *upf3* mutants have increased sensitivity to salt stress, emphasizing the important role that UPF3 and thus proper NMD functioning play in adapting to an abiotic stressor. Similar findings have shown relative increases in NMD-targeted splicing variants following salt stress, further underlining the ability of NMD to react in a stress-responsive manner and raising the possibility that NMD can act as an adaptive mechanism (Drechsel *et al*., 2013). In their analysis of circadian clock genes in *Arabidopsis*, Kwon *et al*. (2014) were able to pinpoint changes in AS leading to NMD-targeted degradation of a select group of these genes following plant exposure to heat and cold stress. These studies, along with our own, add to an emerging body of literature that implicates the role of NMD as a possible mediator of the abiotic stress response.

Our detection method represents a primary, or top-level, assessment of putative NMD targets. Although our full-length reads allow us to directly detect a PTC within an ORF, which strengthens the relevance of our approach, this does not guarantee an isoform is targeted to an NMD pathway or results in the subsequent production of a truncated non-functional protein (Boothby *et al*., 2013). Isoforms with in-frame PTCs may be rescued through the reversible sequestration of splicing intermediates, by which IR-containing isoforms escape cytoplasmic NMD via sequestration into the nucleus where the removal of the retained introns and subsequent release of mature mRNAs occurs (Boothby and Wolniak, 2011). Conversely, studies of *Arabidopsis* NMD mutants have revealed that a small proportion of NMD-sensitive transcripts do not contain the characteristic NMD-inducing in-frame PTCs, indicating that NMD-eliciting features probably encompass more than just in-frame PTCs (Kalyna *et al*., 2012). Although our data present further evidence for an AS-NMD response to environmental stressors, more work needs to be done to examine these processes in finer detail. Detailed expression analysis of NMD factors in *B. napus*, such as UP1, UP2 and UP3 homologues, will offer further insights into how NMD is precisely regulated. Furthermore, similar analyses of splicing factors in *B. napus* could help uncover precisely how NMD-targeted degradation could be regulated via AS and thus help determine exactly how AS-NMD operates in the abiotic stress response within this important crop species.

### Concluding remarks

Overall, our study contributes to a fuller understanding of the complex transcriptional dynamics at play in the allopolyploid *B. napus* in the context of temperature stress. Our use of long-read sequencing has enabled us to assess the dynamic nature of AS and its overall effects on the transcriptome. Through the capture and sequencing of full-length mRNA isoforms we were able to precisely determine how AS activity results in a complex transcriptome, as direct isoform detection removes ambiguities introduced when short reads are reconstructed to resolved isoforms. We documented differential levels of AS in response to heat and cold stress, and across subgenomes, including shifted isoform distributions that may facilitate NMD. Iso-Seq has allowed us to investigate these dynamics with a level of certainty previously unavailable using a short-read RNA-seq approach. Future studies could examine the effects of abiotic stresses on transcript isoform composition in additional tissue/organ types, as well as compare the responses of polyploids and their diploid progenitors using resynthesized lines.

## Supplementary Material

mcaf220_Supplementary_Data

## Data Availability

The Iso-seq data reported in this study were deposited in NCBI’s Sequence Read Archive with accession ID PRJNA1226649.

## SUPPLEMENTARY DATA

Supplementary data are available at *Annals of Botany* online and consist of the following. Figure S1. Flow chart describing the processing of raw isoform sequence data, alignment and the collapsing of redundant isoforms. Figure S2. Genes captured in each condition, showing 32 449 genes in total collected across all three conditions. Figure S3. Distribution of isoforms across structural categories in each condition. FSM: full splice match, ISM: incomplete splice match, NIC: novel in catalogue, NNC: novel not in catalogue. Figure S4. Isoform models of known cold and heat responsive homeologous pairs. (a) COR (cold response) homeologous pair BnaA03g38950 and BnaC03g45990 across the normal and cold conditions. (b) Heat shock protein homeologous pair BnaA06g07260 and BnaCnng18070 across the normal and hot conditions. Figure S5. (a) Proportion of isoforms predicted to undergo NMD across conditions, *n* = 31 953 genes. A ‘likely’ prediction denotes the case in which a termination codon was detected within the ORF, while ‘unlikely’ represents the absence of a premature termination codon detected within the ORF. Frequencies of isoforms in each of the prediction categories varies significantly in response to heat (Fisher’s exact test, *P* = 4.6 × 10^−7^). (b) Across conditions and subgenomes, *n* = 8744 homeologous pairs are shared across all three conditions. Lighter colour represents ‘likely’, and darker colour represents ‘unlikely.’ In each condition the subgenomic origin of a given isoform and its prediction of NMD are independent [Fisher’s exact test, *P* = 0.754 (normal), *P* = 0.287 (cold), *P* = 0.218 (hot)]. Figure S6. RT-PCR verification of alternative splicing events. Shown are five genes with PCR done with reverse transcriptase (RT+) and without reverse transcriptase (RT−) as a negative control to check for genomic DNA contamination. Numbers above the gels are partial gene numbers; see Supplementary Data Table S11 for complete gene numbers. Numbers in red indicate band sizes. The lanes with five bands are size marker lanes. Table S1. Summary of read counts at each filtering step with mapping accuracies. Table S2. Genes and isoforms obtained across the three abiotic conditions. Table S3. Genes and isoforms obtained across the three abiotic conditions, per subgenome. Table S4. Summary of isoform classifications per category across all three conditions. Table S5a. Summary of the top 10 enriched GO terms for the biological process (BP) domain, for the cold and heat responses. Table S5b. Summary of the top 10 enriched GO terms for the molecular function (MF) domain, for the cold and heat responses. Table S5c. Summary of the top 10 enriched GO terms for the cellular compartment (CC) domain, for the cold and heat responses. Table S6. AS event counts and percentages across abiotic conditions per subgenome, *n* = 8744 homeologous gene pairs. Table S7. Summary of the counts of homeologous genes that undergo AS, by condition and subgenome. Table S8a. Summary of the top 5 enriched GO terms for the biological process (BP) domain across homeologous pair categories. Table S8b. Summary of the top 5 enriched GO terms for the molecular function (MF) domain across homeologous pair categories. Table S8c. Summary of the top 5 enriched GO terms for the cellular compartment (CC) domain across homeologous pair categories. Table S9. Counts of homeologous gene pairs for each type of stress responsive isoform ratio category shift. Table S10a. Summary of the top 5 enriched GO terms for the biological process (BP) domain across isoform repertoire shift categories. Table S10b. Summary of the top 5 enriched GO terms for the molecular function (MF) domain across isoform repertoire shift categories. Table S10c. Summary of the top 5 enriched GO terms for the cellular compartment (CC) domain across isoform repertoire shift categories. Table S11. PCR primers
