# Supplementary material for: Transcript isoforms and alternative splicing in polyploid Brassica napus under heat and cold stress
Source: Ann Bot. 2025 Sep 11;137(1):181–95. doi: 10.1093/aob/mcaf220 (PMC12784076; doi:10.1093/aob/mcaf220)
Supplement: mcaf220_Supplementary_Data [file mcaf220_supplementary_data.zip › supplementary figures.docx]

**Supp. Figure 1.** Flow chart describing the processing of raw isoform sequence data, alignment, and the collapsing of redundant isoforms.


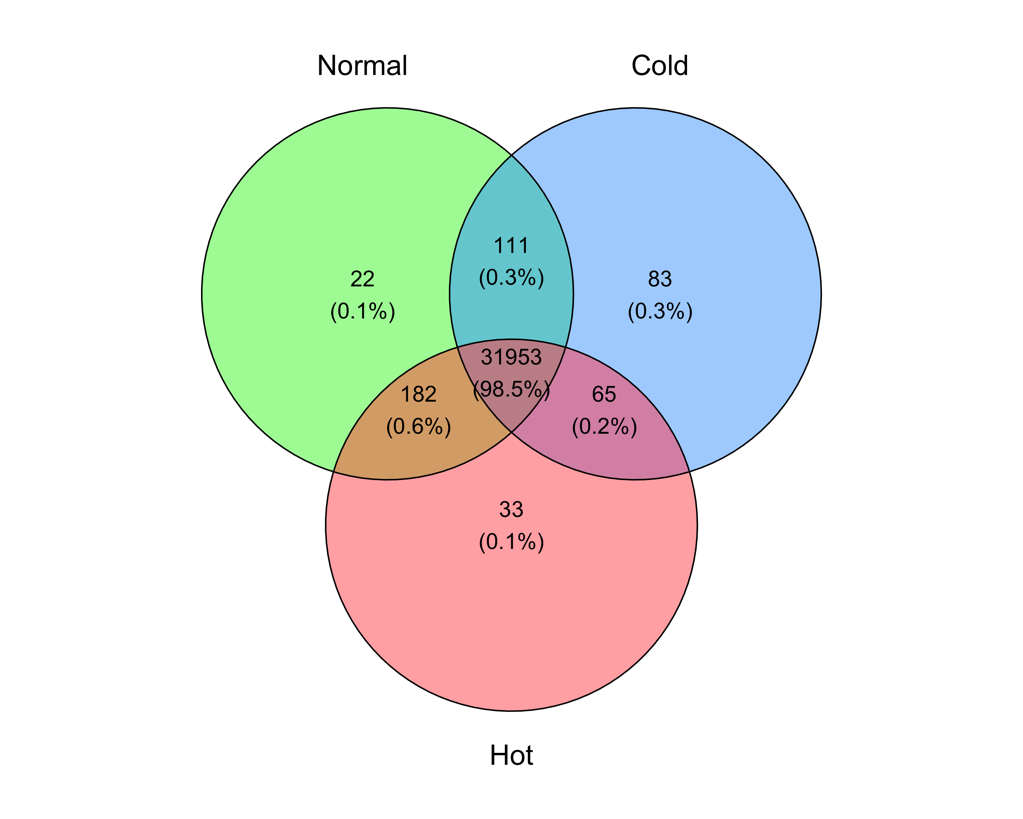


**Supp. Figure 2.** Genes captured in each condition, showing 32,449 total genes collected across all three conditions.


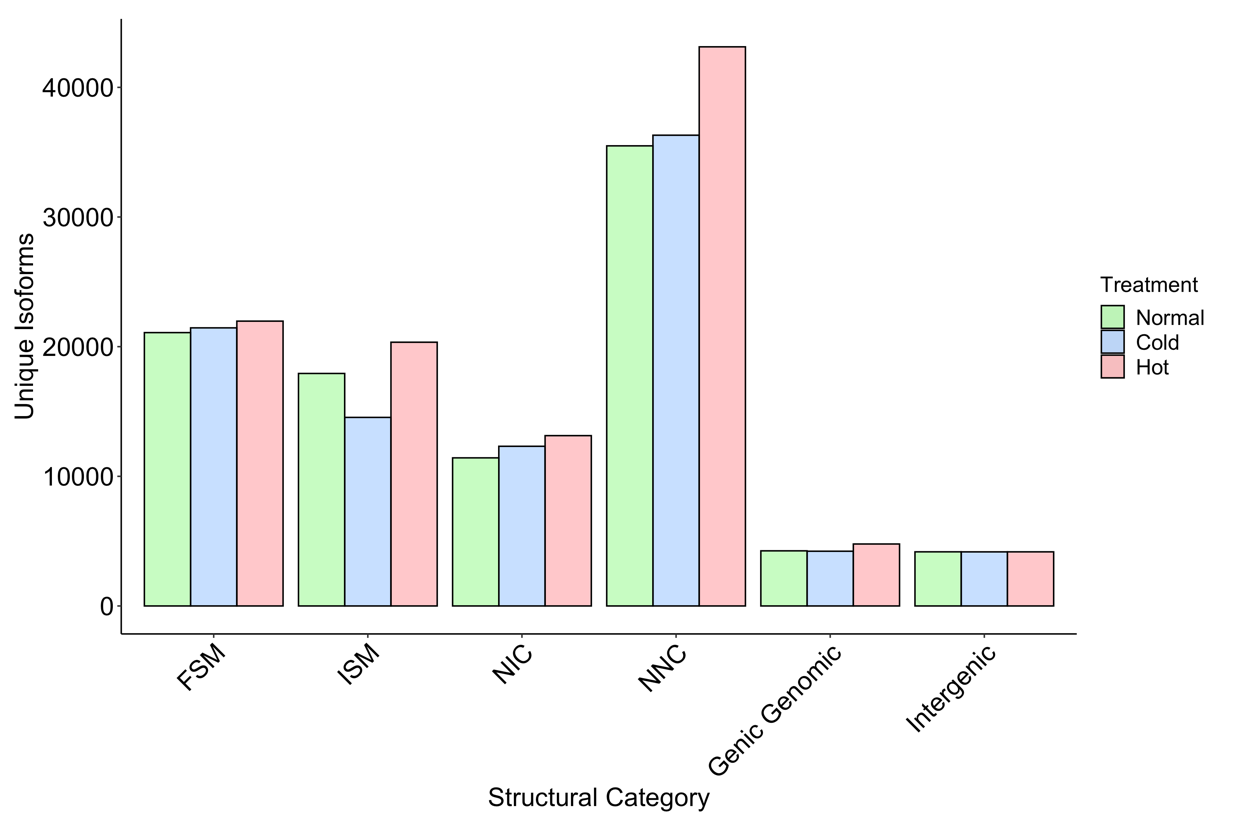


**Supp. Figure 3.** Distribution of isoforms across structural categories in each condition. FSM: full splice match, ISM: incomplete splice match, NIC: novel in catalogue, NNC: novel not in catalogue.

**A**

**B**

**Supp. Figure 4.** Isoform models of known cold and heat responsive homeologous pairs. a) COR (cold response) homeologous pair BnaA03g38950 and BnaC03g45990 across the normal and cold conditions. b) Heat shock protein homeologous pair BnaA06g07260 and BnaCnng18070 across the normal and hot conditions.

*P* = 4.6 x 10^-7^

**Supp. Figure 5** a) Proportion of isoforms predicted to undergo NMD across conditions, n=31,953 genes. A “likely” prediction denotes the case in which a termination codon was detected within the ORF, “unlikely” represents the absence of a premature termination codon detected within the ORF. Frequencies of isoforms in each of the prediction categories varies significantly in response to heat (Fisher’s Exact Test, P=4.6 x 10^-7^). b) Across conditions and subgenomes, n = 8,744 homeologous pairs shared across all 3 conditions. Lighter colour represents “likely”, darker colour represents “unlikely.” In each condition the subgenomic origin of a given isoform and its prediction of NMD are independent (Fisher’s Exact Test, P=0.754 (normal), P=0.287 (cold), P=0.218 (hot)).

05140 30110 24200 30430 26540

RT + - + - + - + - + -


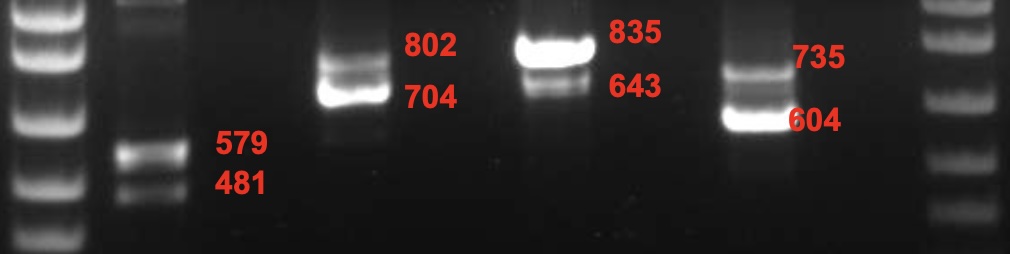

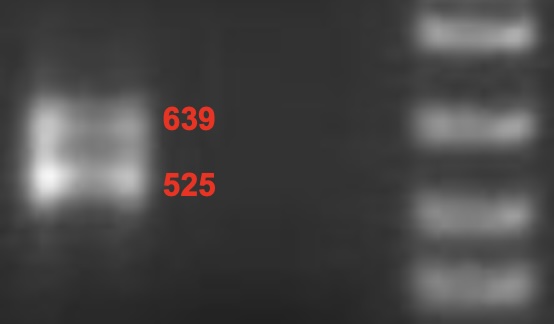


**Supp. Figure 6.** RT-PCR verification of alternative splicing events. Shown are five genes with PCR done with reverse transcriptase (RT+) and without reverse transcriptase (RT-) as a negative control to check for gDNA contamination. Numbers above the gels are partial gene numbers; see supplemental table 11 for complete gene numbers. Numbers in red indicate band sizes. The lanes with 5 bands are size marker lanes.
