# Supplementary material for: Transcript isoforms and alternative splicing in polyploid Brassica napus under heat and cold stress
Source: Ann Bot. 2025 Sep 11;137(1):181–95. doi: 10.1093/aob/mcaf220 (PMC12784076; doi:10.1093/aob/mcaf220)
Supplement: mcaf220_Supplementary_Data [file mcaf220_supplementary_data.zip › supplementary tables.docx]

**Supp. Table 1.** Summary of read counts at each filtering step with mapping accuracies

|  | **Subreads** | | | | |
| --- | --- | --- | --- | --- | --- |
| *Replicate* | *Normal* | *Cold* | | | *Hot* |
| 1 | 14,848,397 | 13,384,860 | | | 13,626,459 |
| 2 | 15,121,875 | 11,571,192 | | | 13,805,623 |
| 3 | 15,913,397 | 8,557,455 | | | 13,201,831 |
|  | **CCS reads** | | | | |
| 1 | 438,871 | 403,062 | | | 410,997 |
| 2 | 472,682 | 336,920 | | | 372,842 |
| 3 | 426,020 | 189,787 | | | 419,008 |
|  | **FLNC reads** | | | | |
| 1 | 375,265 | 356,445 | | | 358,491 |
| 2 | 412,514 | 296,433 | | | 329,857 |
| 3 | 361,750 | 169,571 | | | 354,826 |
|  | **Mapping Accuracy (%)** | | | | |
|  | 99.92 | | 99.94 | 99.91 | |
|  | **Collapsed Isoforms** | | | | |
|  | 131,749 | | 125,266 | 156,029 | |

**Supp. Table 2.** Genes and isoforms obtained across the three abiotic conditions.

|  | **Normal** | **Cold** | **Hot** | |
| --- | --- | --- | --- | --- |
| Unique Genes | 32,268 | 32,212 | | 32,233 |
| Unique Isoforms | 98,861 | 96,965 | | 111,976 |
| Isoforms per gene | 3.06 | 3.02 | | 3.47 |

**Supp. Table 3.** Genes and isoforms obtained across the three abiotic conditions, per subgenome.

|  | **Normal** | | **Cold** | | **Hot** | |
| --- | --- | --- | --- | --- | --- | --- |
| Subgenome | A_T_ | C_T_ | A_T_ | C_T_ | A_T_ | C_T_ |
| Genes | 8,744 | 8,744 | 8,744 | 8,744 | 8,744 | 8,744 |
| Isoforms | 30,623 | 31,013 | 30,702 | 30,939 | 35,058 | 35,388 |
| Isoforms/Gene | 3.50 | 3.55 | 3.51 | 3.54 | 4.01 | 4.05 |

**Supp. Table 4.** Summary of isoform classifications per category across all three conditions.

|  | **Normal** | **Cold** | **Hot** |
| --- | --- | --- | --- |
| FSM | 21,083 (22.1%) | 21,450 (22.8%) | 21,970 (20.2%) |
| ISM | 17,933 (18.8%) | 14,545 (15.5%) | 20,342 (18.7%) |
| NIC | 11,424 (12.0%) | 12,318 (13.1%) | 13,138 (12.1%) |
| NNC | 35,487 (37.3%) | 36,308 (38.7%) | 43,124 (39.7%) |
| Genic Genomic | 4,254 (4.5%) | 4,224 (4.5%) | 4,781 (4.4%) |
| Other | 5,107 (5.4%) | 5,089 (5.4%) | 5,215 (4.8%) |
| Total | 95,227 | 93,934 | 108,570 |

**Supp. Table 5a.** Summary of the top 10 enriched GO terms for the biological process (BP) domain, for the cold and heat responses.

**Supp. Table 5b**. Summary of the top 10 enriched GO terms for the molecular function (MF) domain, for the cold and heat responses.

**Supp. Table 5c.** Summary of the top 10 enriched GO terms for the cellular compartment (CC) domain, for the cold and heat responses.

**Supp. Table 6.** AS event counts and percentages across abiotic conditions per subgenome, n=8,744 homeologous gene pairs.

|  | **Normal** | **Cold** | **Hot** |
| --- | --- | --- | --- |
| Total Genes | 31,953 | 31,953 | 31,953 |
| AS Events | 26,683 | 27,579 | 33,381 |
| AS Genes | 10,384 | 10,294 | 12,187 |
| AS events/gene | 2.57 | 2.68 | 2.74 |

**Supp. Table 7.** Summary of the counts of homeologous genes that undergo AS, by condition and subgenome

|  | Normal (*A_T_)* | Normal *(C_T_)* | Cold *(A_T_)* | Cold *(C_T_)* | Hot *(A_T_)* | Hot *(C_T_)* |
| --- | --- | --- | --- | --- | --- | --- |
| Total Genes | 8,744 | 8,744 | 8,744 | 8,744 | 8,744 | 8,744 |
| AS Events | 10,925 | 11,402 | 11,648 | 11,925 | 13,704 | 14,408 |
| AS Genes | 4,344 | 4,476 | 4,395 | 4,472 | 5,132 | 5,292 |
| AS events/gene | 2.51 | 2.55 | 2.65 | 2.67 | 2.67 | 2.72 |

**Supp. Table 8a.** Summary of the top 5 enriched GO terms for the biological process (BP) domain across homeologous pair categories.

******Supp. Table 8b.** Summary of the top 5 enriched GO terms for the molecular function (MF) domain across homeologous pair categories.

**Supp. Table 8c.** Summary of the top 5 enriched GO terms for the cellular compartment (CC) domain across homeologous pair categories.

**Supp. Table 9.** Counts of homeologous gene pairs for each type of stress responsive isoform ratio category shift.

| **Category Shift**  **(Normal 🡪 Stress)** | **Pairs (Cold Response)** | **Pairs (Heat Response)** |
| --- | --- | --- |
| C_T_ > A_T_ 🡪 C_T_ > A_T_ | 706 (8.1%) | 724 (8.3%) |
| C_T_ > A_T_ 🡪 A_T_ = C_T_ | 965 (11.0%) | 1028 (11.6%) |
| C_T_ > A_T_ 🡪 A_T_ > C_T_ | 298 (3.4%) | 217 (2.5%) |
| A_T_ = C_T_ 🡪 C_T_ > A_T_ | 1033 (11.8%) | 953 (10.9%) |
| A_T_ = C_T_ 🡪 A_T_ = C_T_ | 2994 (34.2%) | 3139 (35.9%) |
| A_T_ = C_T_ 🡪 A_T_ > C_T_ | 965 (11.0%) | 900 (10.3%) |
| A_T_ > C_T_ 🡪 C_T_ > A_T_ | 253 (2.9%) | 220 (2.5%) |
| A_T_ > C_T_ 🡪 A_T_ = C_T_ | 902 (10.3%) | 925 (10.6%) |
| A_T_ > C_T_ 🡪 A_T_ > C_T_ | 628 (7.2%) | 638 (7.3%) |

**Supp. Table 10a.** Summary of the top 5 enriched GO terms for the biological process (BP) domain across isoform repertoire shift categories.

**Supp. Table 10b.** Summary of the top 5 enriched GO terms for the molecular function (MF) domain across isoform repertoire shift categories.

**Supp. Table 10c**. Summary of the top 5 enriched GO terms for the cellular compartment (CC) domain across isoform repertoire shift categories.

**Supp. Table 11.** PCR primers

| Gene | Condition | F_primer | R_primer | AS  type | Putative function |
| --- | --- | --- | --- | --- | --- |
| BnaA07g05140D | Hot | TGGTCAAGTATGATGTGCTACC | GCACATGCTTAACATCCATTGT | IR | Unknown |
| BnaC02g30110D | Hot | GGTGTTAATCTCAGGGAAGAGG | CGACCACCAACTCCAGTAAA | IR | Unknown |
| BnaA04g24200D | Hot | AGCAGGCTGAGATCTTCTTTC | CGAGCTGCATTTGCTCAATC | IR | Unknown |
| BnaA07g30430D | Cold | TGGTTCTTCTGATGTGGTTCTT | AGCTCTGGTCTTGAACTTTCTT | IR | Hypersensitive to ABA 1 |
| BnaC05g26540D | Hot | GGAAGCAACAGAGGAGAGATTAG | CCACCTCAAGCAATCCAATAAAC | IR | Unknown |
